# Establishment of a sticky, large, oval-shaped thrombocyte cell line from tree frog as an ancestor of mammalian megakaryocytes

**DOI:** 10.1186/s40064-015-1237-7

**Published:** 2015-08-25

**Authors:** Kenkichi Sugimoto

**Affiliations:** Department of Cell Science, Faculty of Graduate School of Science and Technology, Niigata University, Nishi-ku, Ikarashi-2, Niigata 950-2181 Japan

**Keywords:** Thrombocyte, Amphibian, Large cell, Adhesion, Integrin alpha IIb

## Abstract

Maintenance of blood vessels is important for homeostasis. Many types of cells and cytokines are involved in angiogenesis and blood vessel repair. In mammals, platelets, which are produced from megakaryocytes, play a major role in hemostasis. Other vertebrates have no platelets in their bloodstream. In these animals, thrombocytes aggregate to form a thrombus. Therefore, I established a frog hematopoietic cell line to elucidate the mechanism of hematopoiesis in this species. The frog-derived thrombocytic cell line was established from a long-term bone marrow culture of *Hyla japonica* and was designated as a frog-derived unique hematopoietic non-adherent (FUHEN) cell line. The FUHEN cells had unique characteristics in that they proliferated in suspension culture without adherence to the culture flask, and the shapes of the FUHEN cells changed drastically to become very large ovals with growth. These cells reached more than 40 µm in length and had multi-lobed nuclei. The FUHEN cells expressed CD41, a specific surface marker of thrombocytes. These results indicated that the FUHEN cells were thrombocytes. Deprivation of divalent ions quickly induced adherence of the cells to the petri dish. This characteristic may be important for hemostasis. Furthermore, some of the FUHEN cells survived at 16 °C for 1 month and re-established proliferation when the cells were moved to 28 °C. Taken together, this new thrombocytic frog cell line, as an ancestor of mammalian megakaryocytes, could provide useful material to study the functions of thrombocytes and the hemostasis mechanism of amphibians.

**Electronic supplementary material:**

The online version of this article (doi:10.1186/s40064-015-1237-7) contains supplementary material, which is available to authorized users.

## Background

Hibernation is an important physiological phenomenon. Many species of amphibians as well as some species of mammals hibernate in winter. However, the molecular control mechanism of hematopoiesis during hibernation in these hibernators has not been completely elucidated. One of the reasons for the delay in this field is that there is no suitable established hematopoietic cell line for hibernators. Frogs are among the most famous hibernators of the poikilothermic animals. Some frog cell lines have been established (Fukui et al. 1992; Okumoto et al. 1995), although these cells are non-hematopoietic.

The maintenance of blood vessels and the regulation of hematopoiesis are very important for vertebrate homeostasis. Angiogenesis and remodeling of capillaries occur at all times. Many types of cells and many types of cytokines are involved in both angiogenesis and wound healing. Above all, the key factors are vascular endothelial growth factors (VEGFs) (Johnson and Wilgus 2014). However, the hemostasis system also plays an important role in blood vessel maintenance. Rapid hemostasis is needed to both prevent blood loss and repair blood vessels. In mammals, platelets produced from megakaryocytes play a pivotal role in hemostasis. This aggregation cascade is initiated by the attachment of von Willebrand factor (vWF) to collagen in the injured part of the blood vessel (Reininger 2008; Lenting et al. 2012). Subsequently, platelets attach to the A1 domain of vWF through glycoprotein Ib/IX (GPIb/IX) on the cell membrane. Thrombus formation then occurs as a consequence of activation and aggregation (Angiolillo et al. 2010). Furthermore, in addition to biological factors, physical stress is involved in hemostasis. Shear stress also activates platelets to form thrombi (Dopheide et al. 2002; Reininger et al. 2006; Maxwell et al. 2007; Jackson 2007).

In contrast to mammals, fish, amphibians, reptiles and birds have no platelets in their bloodstream (Michelson 2013); this suggests that there are no cells similar to the “megakaryocytes” of mammals, which produce platelets in these animals. These non-mammals have thrombocytes in their bloodstream instead of megakaryocytes, and these cells aggregate to form a thrombus in hemostasis. Thus, the activation mechanism of thrombocytes must be tightly regulated in blood vessels. Using zebrafish, the function and characterization of thrombocytes (Jagadeeswaran et al. 1999; Kim et al. 2010; Khandekar et al. 2012) and their development (Lin et al. 2005) have been studied. However, the molecular mechanisms of thrombus formation have not been precisely elucidated. A suitable thrombocytic cell line could be useful to increase the understanding of thrombus formation. It is well known that many species of wild amphibians, such as salamanders, newts and frogs, hibernate in winter in Japan. Japanese tree frogs (*Hyla japonica*) exist throughout the Niigata prefecture, and almost all frogs hibernate in winter (from mid-November to early April of the next year) (Sugimoto and Jiang 2008). Thus, tree frogs are suitable animals to study the relationship between hematopoiesis and hibernation, and the establishment of a hematopoietic cell line could be useful in the study of the hematopoietic system of wild tree frogs in vitro.

Here, I report the establishment of a new tree frog thrombocytic cell line. Based on the observation of hematopoiesis in the bone marrow of tree frogs, a frog-derived unique hematopoietic non-adherent (FUHEN) cell line was established from a long-term bone marrow culture (LTBMC). This FUHEN cell line has unique characteristics. The FUHEN cells proliferated in suspension culture without adherence to the culture flask, and the shapes of the FUHEN cells changed drastically by growing into very large ovals, reaching more than 40 µm in length, with multi-lobed nuclei. The FUHEN cells expressed CD41, which is a specific surface marker of thrombocytes. Deprivation of divalent ions quickly induced adherence of the cells to a petri dish. Furthermore, some of the FUHEN cells could be sustained at 16 °C for 1 month, and proliferation returned when the cells were moved to 28 °C. Taken together, this new thrombocytic cell line could provide useful material as an ancestor of mammalian megakaryocytes to study the function of thrombocytes and the hemostasis mechanism of amphibians.

## Results

### Hematopoiesis in the bone marrow of tree frogs

**Fig. 1:**
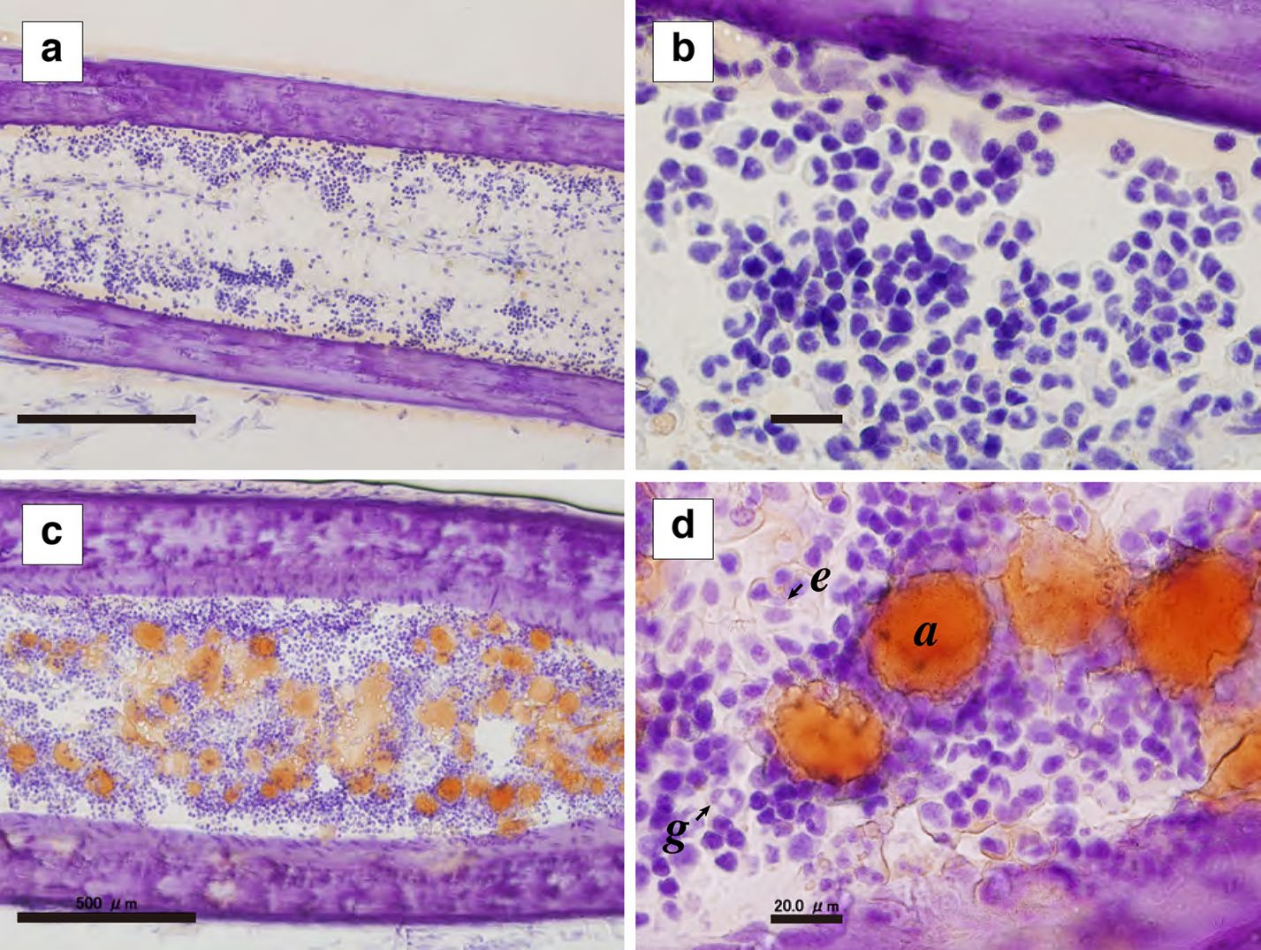
Seasonal changes in hematopoiesis in the bone marrow. Frozen sections of frog tibias obtained in July (**a**, **b**) or in February (**c**, **d**) were double-stained with Oil Red O and hematoxylin*. Scale bars* indicate 500 µm (**a**, **c**) or 20 µm (**b**, **d**). Adipocytes (*a*), granulocytes (*g*) and erythrocytes (*e*) are indicated

To clarify the role of bone marrow in wild tree frogs, thin sections of tibia were stained (Fig. 1). In the sample from July, many nucleated cells were present in the region near the endosteum rather than the core of the bone marrow, and they formed hematopoietic loci. Many nucleated cells were granulocytes, and there were few erythrocytes; thus, the bone marrow of tree frogs consisted of myeloid hematopoietic tissue, indicating that the region near the endosteum was the niche for hematopoiesis in tree frogs. Interestingly, more nucleated cells existed in the February sample than the July sample. Obvious accumulation of a large amount of fat in the adipocytes was also observed, indicating that the bone marrow of tree frogs might act as an energy reservoir during hibernation.

### Establishment of the FUHEN cell line

**Fig. 2:**
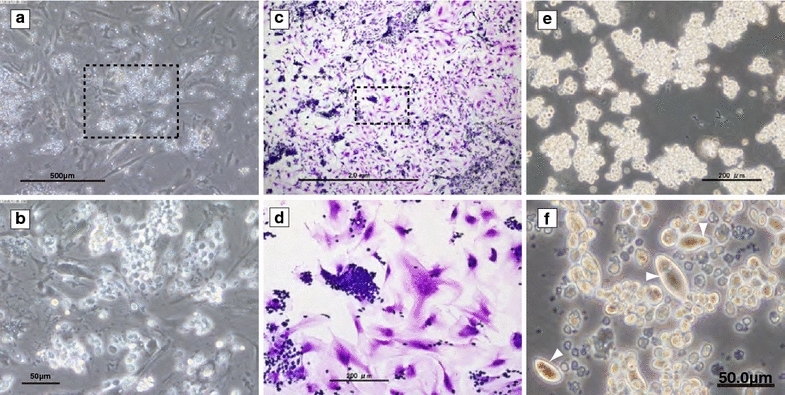
FUHEN cells in culture. The frog LTBMC. Large adherent stromal cells and hematopoietic cells were observed (phase contrast, **a** and **b**). The *dashed square* in **a** is magnified in **b**. The LTBMC was stained with May-Grünwald Giemsa (**c**, **d**). The *dashed square* in **c** is magnified in **d**. *Scale bar* indicates 2 mm (**c**) or 200 µm (**d**). Non-adherent cells in the LTBMC were selected, and the FUHEN cell line was established. The proliferating FUHEN cells formed large clumps (**e**) after 2 weeks at 28 °C. The clump size was more than 200 µm. After 4 weeks at 28 °C, huge oval-shaped cells were observed. Collar structures were observed in the oval-shaped cells (**f**). The *white triangles* represent the positions of the collar structures in the cells. *Scale bar* indicates 200 µm (**e**) or 50 µm (**f**)

A hematopoietic frog cell line had not been established previously. An LTBMC of tree frog cells was started because histochemical analysis revealed the existence of many hematopoietic cells in the bone marrow. After 3 weeks, the LTBMC achieved steady conditions: both hematopoietic and stromal cells proliferated in the culture, and many hematopoietic foci, in which a large number of hematopoietic cells adhered to the stromal cells, were observed (Fig. 2a–d). The shapes of the stromal cells were flat and elongated, similar to murine stromal cells. However, many spherical or oval-shaped hematopoietic cells existed in the flask. After X-ray irradiation, the nucleated cells, but not the erythrocytes, started to proliferate significantly in suspension culture without any factors or stromal cells. Then, these cells were selected, cloned and designated as the FUHEN cell line. The FUHEN cells proliferated in the suspension culture without adherence to the culture flask and were also independent from stromal cells; however, the smaller FUHEN cells attached to each other and formed clumps when they proliferated. The clump size was larger than 200 µm (Fig. 2e). In contrast, clump formation was rare among large FUHEN cells. The ordinal diameter of the FUHEN cells was approximately 15 µm, and the shape was usually spherical or slightly oval. Giemsa staining revealed little cytosol and large nuclei. Interestingly, the sizes of the FUHEN cells changed drastically based on the rate of growth. After 4 weeks of passage at 28 °C, some of these cells grew to become oval-shaped cells, with long diameters greater than 40 µm (Fig. 2f). Furthermore, a collar structure was clearly observed in the oval-shaped large FUHEN cells (Fig. 2f). With standard maintenance, the FUHEN cells were passaged every 3 weeks by splitting 1/3 with fresh medium and incubating at 28 °C. Therefore, the culture included both large and normal-sized cells. The FUHEN cells proliferated constantly, and it was possible to store the cells in liquid nitrogen.

### Temperature-sensitive growth of FUHEN cells

**Fig. 3:**
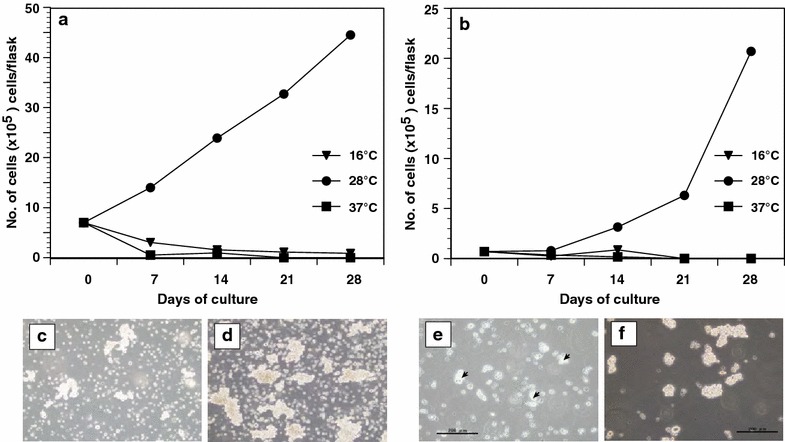
Temperature-sensitive growth of FUHEN cells. Growth of FUHEN cells was analyzed at 16, 28 and 37 °C (**a**, **b**). *Symbols* indicate the culture temperature (*circle* 28 °C, *square* 37 °C, *triangle* 16 °C). The seeded cell number was either 7 × 10^4^ cells/flask (**a**), or 7 × 10^5^ cells/flask (**b**). The proliferating cells at 28 °C after 1 week (**c**) or 3 weeks (**d**). The surviving cells at 16 °C after 4 weeks are indicated (**e**, *arrow head*). The cells resumed proliferation when the temperature was shifted to 28 °C after 2 weeks (**f**)

Growth of FUHEN cells under various temperature conditions was analyzed (Fig. 3a, b). The FUHEN cells proliferated at 28 °C and formed clumps (Fig. 3c, d). The doubling time of FUHEN cells at 28 °C was estimated at 197 h. However, the FUHEN cells could not survive at 37 °C (Fig. 3a, b); all of the FUHEN cells died within 2 weeks at 37 °C. A temperature of 16 °C was not suitable for proliferation because the number of FUHEN cells decreased gradually, although some cells survived after culture at 16 °C for 4 weeks (Fig. 3e). However, the cells began to proliferate and small clumps were observed when the culture flask was shifted from 16 to 28 °C after 2 weeks (Fig. 3f). Thus, the FUHEN cells could survive at 16 °C for at least 4 weeks. Furthermore, surprisingly, some of the FUHEN cells could survive at 28 °C for more than 5 months without the medium being changed (Additional file 1: Fig. S1), and these surviving cells began to proliferate when the cells were suspended in fresh medium (data not shown). However, all the cells died after 1 year without the medium being changed (data not shown). Although the sustainability of the FUHEN cells was not perfect under the severe culture conditions described above, it was stronger than a mammalian cell line.

### Fine structure of the FUHEN cells

**Fig. 4:**
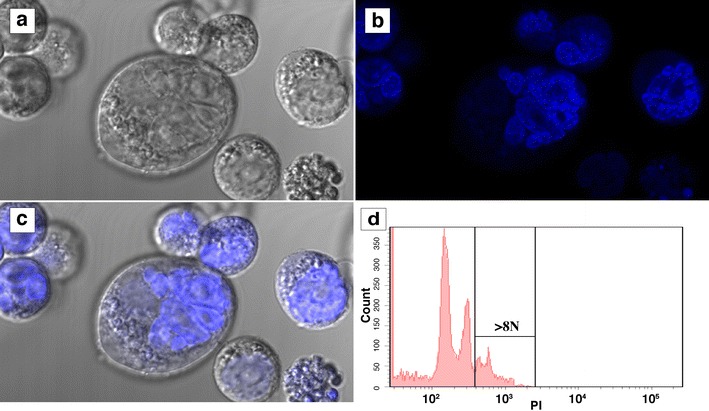
Fine structure of the FUHEN cells. The FUHEN cells were stained with DAPI and observed with a confocal microscope (**a** phase contrast, **b** fluorescence, **c** merged). The nuclear phase of the proliferating FUHEN cells was analyzed by FACS (**d**). Additional file 2: Movie S1a and S1b were taken of the same section as in **b**

DAPI (4′,6-diamidino-2-phenylindole) staining revealed multi-lobulated large nuclei in the FUHEN cells. The nuclei were more than 20 µm in diameter and were located at one end of the cell (Fig. 4a–c; Additional file 2: Movies S1a, S1b, S2a, S2b). One ring-like nuclear structure was observed in a small cell, and a multi-ring-like nuclear structure was observed in a very large cell. Nuclear phase analysis by FACS revealed that 14.2 % of the cells had ploidies of more than 8 N (Fig. 4d). These results indicated that the large FUHEN cells were a thrombocytic cell line with a multi-nuclear phase.

### Apparatus-dependent adhesion of FUHEN cells

**Fig. 5:**
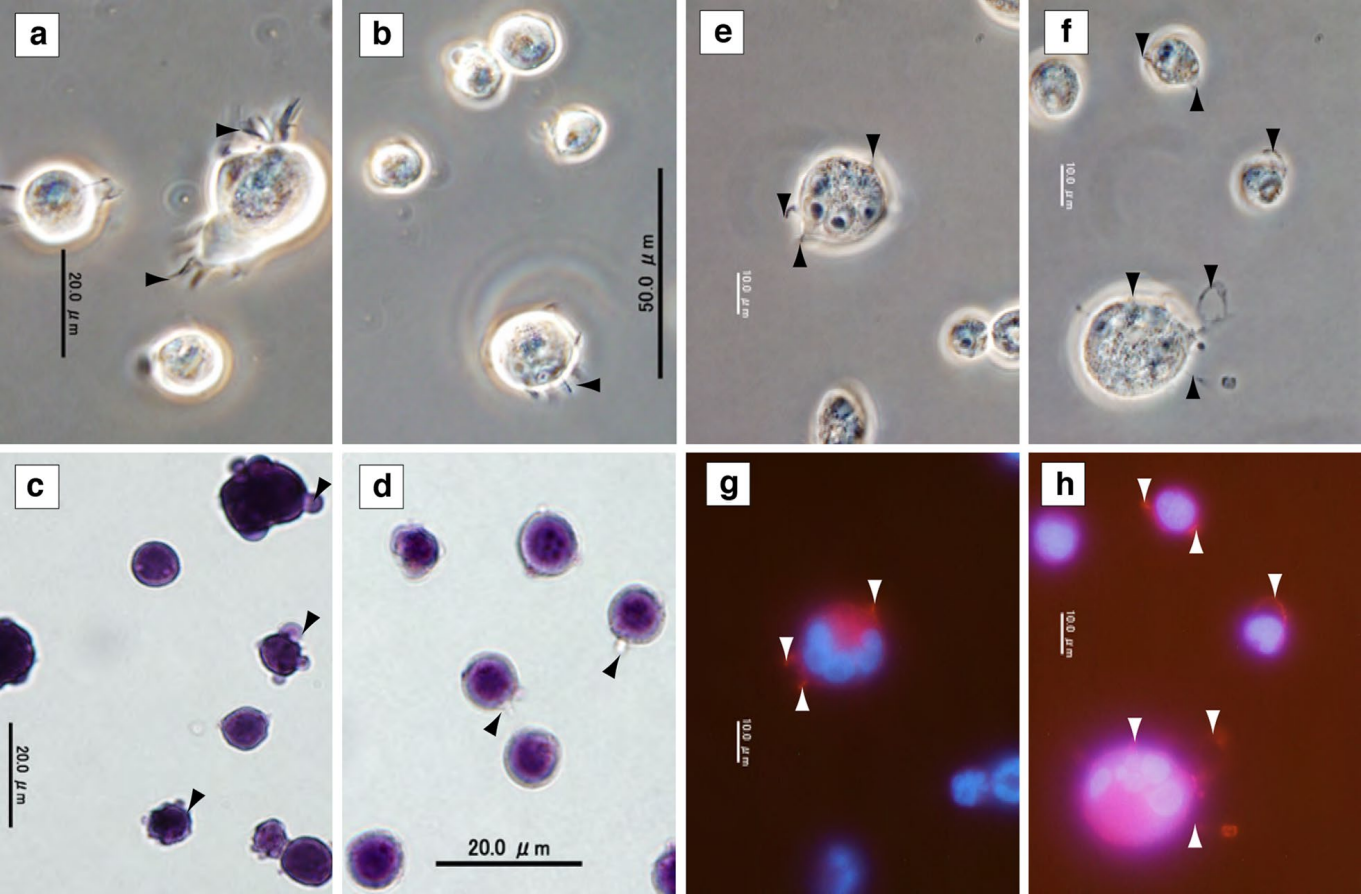
Apparatus-dependent adhesion of FUHEN cells. The FUHEN cells were washed and suspended in EDTA-PBS and seeded in a petri dish. Adherent FUHEN cells (**a**, **b**, phase contrast) (**c**, **d**, HE staining). Fixed cells were stained with the actin stain 555 Fluorescent Phalloidin and DAPI (**e**, **f** phase contrast, **g**, **h** merged photograph). *Black* and *white triangles* indicate the adherent apparatus

The FUHEN cells did not adhere to the tissue culture flask/dish with normal passage. When the cells were suspended in 10 % HS-αMEM, none of the cells adhered to the petri dishes (Table 1). However, when the cells were suspended in PBS or EDTA-PBS, almost all of the cells attached to the petri dishes within 3 min. Because the adhesion was strong, it was hard to remove the cells from dishes with pipetting. This adhesion was prevented by the addition of HS, bovine serum albumin (BSA), and Ca^2+^ and Mg^2+^. Observation of adherent cells revealed that they protruded above the adhesion apparatus (Fig. 5).

**Table 1:** Adhesion test

Solution	No. of adhered cells/area (mean ± standard deviation)
10 % HS-αMEM (control)	0 ± 0
0.02 % EDTA-PBS	43.3 ± 6.9*
PBS	39.8 ± 9.6*
PBS + MgCl_2_ + CaCl_2_	0 ± 0
10 % HS-PBS	2.8 ± 2.4
10 % HS-0.02 % EDTA-PBS	0.5 ± 1.0

The FUHEN cells were washed two times with 1 % HS-αMEM, and were suspended with various solutions, respectively. Then the cells were seeded in four petri dishes (Falcon 1008) at a density of 3 × 10^4^ cells/dish. After 5 min of incubation at room temperature, the dishes were washed two times with PBS and fixed with 70 % EtOH for 3 h. Then, the cells were stained with hematoxylin or May-Grünwald Giemsa, and photographs were taken. The number of attached cells per area was counted. One-way ANOVAs and multiple comparison tests (Tukey–Kramer’s HSD test) were carried out

* Means significant (*p* < 0.05) difference compared to the control

### Partial cloning of *cd41*

**Fig. 6:**
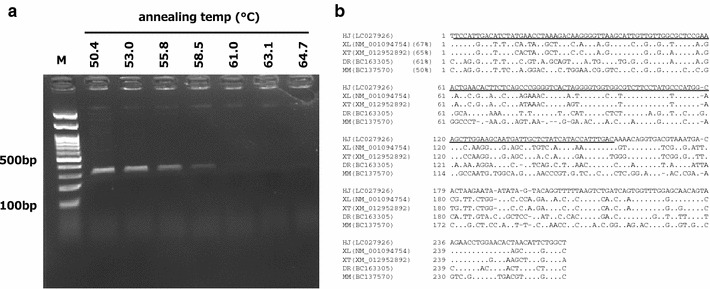
*cd41* expression in FUHEN cells. The partial coding sequence of *cd41* (integrin alpha 2b) was cloned (NCBI accession no. LC027926). Degenerate PCR primers amplified a DNA fragment with a predicted size of 312 bp (**a**). The alignment of corresponding *cd41* sequences in *Hyla japonica* (LC027926), *Xenopus laevis* (NM_001094754), *Xenopus* (*Silurana*) *tropicalis* (XM_012952892), *Danio rerio* (BC163305) and *Mus musculus* (BC137570) is indicated (**b**). The alignment was carried out using GENETYX-MAC software (Genetyx Co. Ltd., Tokyo, Japan). The cloned DNA fragment (262 bp) had 67 % homology to *Xenopus laevis* alpha 2b. The *underlined* amino acids indicate the integrin alpha superfamily domain

Partial cloning of the *cd41* gene of FUHEN cells was achieved using degenerate PCR primers. The degenerate PCR primers were designed according to a previous report (Lin et al. 2005). The degenerate primers successfully amplified DNA from cDNA derived from the FUHEN cells (Fig. 6a). The amplified fragment was then cloned into a cloning vector, and the sequence was determined (NCBI accession no. LC027926). The alignment of the corresponding *cd41* sequences among several species is shown (Fig. 6b). The cloned sequence demonstrated 67 % homology with *Xenopus laevis* integrin alpha 2b (accession no. NM_001094754.1). The deduced amino acid sequence contained the integrin alpha superfamily domain. These data indicated that FUHEN cells were thrombocytes.

## Discussion

### Hematopoiesis in tree frog bone marrow

One of the physiological features during hibernation is hypometabolism. The regulatory mechanism of hypometabolism was reviewed by Storey (2015). Briefly, the reversible phosphorylation of two key proteins (i.e., eIF4E and 4E-BP1) regulates initiation, and RNA polymerase II contributes to the suppression of metabolism (van Breukelen et al. 2004; Storey and Storey 2007). Furthermore, epigenetic regulatory mechanisms also participate in the regulation of hypometabolism. For instance, the activity of histone deacetylase has been shown to be increased in hibernating ground squirrels, reducing both acetylated histone H3 (Lys 23) and phosphorylated histone H3 (Ser10) (Storey 2015; Morin and Storey 2006; Biggar and Storey 2014), which mediate chromatin packaging. Chromatin packaging suppressed gene expression, and, as a consequence, the metabolic rate in hibernating animals was decreased.

On the other hand, it has been reported that the most complete hematopoietic site of adult Xenopus are found in the sub-capsular region of the liver (Hadji-Azimi et al. 1987), where various cell lineages are produced. However, the bone marrow has been reported to be the main site of differentiation of neutrophilic granulocytes but not the main site of hematopoiesis. In addition, recent histological research using antibodies against Xenopus thrombocytes demonstrated the existence of thrombocytes in both the hepatic sinusoids and splenic red pulp (Tanizaki et al. 2015). In contrast, it was reported in another study that the thrombocytic lineage occurred near the sinusoids of the bone marrow in bullfrogs (de Abreu Manso et al. 2009). Furthermore, it has been reported that committed Xenopus macrophage precursors exist in the bone marrow rather than the peripheral liver (Grayfer and Robert 2013). In contrast to Xenopus, which is usually bred under steady temperature conditions, many species of wild frogs in Japan live in severe environmental temperatures that can vary from −10 to 35 °C. *Hyla japonica* is an abundant wild tree frog in Japan, and this frog hibernates in winter (Sugimoto and Jiang 2008). However, hematopoiesis in tree frogs during hibernation, including whether the bone marrow is the hematopoietic tissue, has not yet been precisely studied. Therefore, this study was initiated to clarify hematopoiesis in bone marrow during hibernation.

Here, histological analysis of wild tree frogs demonstrated more potent hematopoiesis in the bone marrow in February samples compared to July samples and also demonstrated that hematopoiesis occurred in the near-endosteal region rather than in the bone marrow core (Fig. 1). In mammals, the endosteal region is important for hematopoiesis because it forms the niche for hematopoiesis (Arai et al. 2009). Thus, these results indicated that the endosteal region of tree frogs, in addition to mammals, was also important for hematopoiesis. Significant accumulation of fat in the adipocytes suggested that the adipocytes supplied the energy for hematopoiesis during hibernation. Because it has been reported that cold stress up-regulates the expression of myelopoiesis-related factors in zebrafish (Kulkeaw et al. 2010), some myelopoiesis-related factors in wild tree frogs also must be up-regulated under cold stress. Taken together, these data suggest that the bone marrow of wild tree frogs is an important hematopoietic tissue and that its hematopoietic capacity is dynamically regulated according to the environmental temperature.

### Establishment of an amphibian thrombocytic cell line

The method for generating an LTBMC (Allen and Dexter 1982; Whitlock and Witte 1982) was also useful for the establishment of a hematopoietic cell line in amphibians. Seasonal changes in gonadal steroids have been reported in bullfrogs (Licht et al. 1983), and testosterone has also been reported to be effective for the maintenance of murine stem cells because it induces the expression of certain cytokines in stromal cells (Nakayama et al. 2006); therefore, testosterone was used in the LTBMC instead of hydrocortisone. The appearance of the frog LTBMC was almost the same as that of murine LTBMC (Fig. 2), in which large stromal cells and hematopoietic cells co-existed. In murine LTBMC, lethal X-ray irradiation is toxic to proliferating cells; as a consequence, only stromal cells survive after irradiation. Interestingly, although the precise reason was not clear, not only the stromal cells but also the hematopoietic cells proliferated in the frog LTBMC after irradiation. Some of these proliferating hematopoietic cells were uniquely spindle-shaped, which is one of the characteristics of thrombocytes. Therefore, I cloned these proliferating hematopoietic cells, and ultimately, the FUHEN cell line was established.

In adult *Xenopus laevis*, hematopoietic cells have been classified into five lineages: erythrocytes, lymphocytes, granulocytes, monocytes and thrombocytes. The thrombocytes have been tentatively classified into four developmental stages, including prothromboblasts, thromboblasts, young thrombocytes and mature thrombocytes, and they are smaller depending on the developmental stage (Hadji-Azimi et al. 1987). No hematopoietic cell lines, including thrombocytic cell lines, have been established from *Xenopus laevis* until now. The FUHEN cell line was derived from bone marrow and was classified as a thrombocytic cell line. The characteristics of FUHEN cells, such as their unique cell shape, large nucleus, cell size (Fig. 2e, f), polyploid nuclei (Fig. 4d) and sticky nature (adherence property; Table 1), indicated that this cell line was thrombocytic in nature. Because the large FUHEN cells were grown from small FUHEN cells, the developmental steps of thrombocytes might be different from those of innate thrombocytes of *X. laevis* (Hadji-Azimi et al. 1987).

CD41 (glycoprotein αIIb) is one of the major markers of megakaryocytes and thrombocytes (Tanizaki et al. 2015; Finkielsztein et al. 2015). Studies that identified and characterized thrombocytes in zebrafish reported that thrombocytes represent the hemostatic homolog of mammalian platelets (Jagadeeswaran et al. 1999). Analysis of thrombocyte development in zebrafish, including cloning of both the *cd41* and thrombopoietin receptor (c-MPL) genes, demonstrated that CD41 expression was correlated with thrombocyte maturation (Lin et al. 2005). Furthermore, knockdown of CD41 via the morpholino injection of alphaIIb in vivo inhibited the aggregation activity of thrombocytes (Kim et al. 2010). Thus, CD41 is closely correlated with the development and function of thrombocytes. Although *Xenopus tropicaliscd41* has been logged in public databases (NCBI accession no. BC166262), *Hyla japonica cd41* has not been cloned. Therefore, the gene fragment from *Hyla japonica* was cloned. The FUHEN cells expressed CD41, and part of *cd41* was cloned (Fig. 6) (NCBI accession no. LC027926). The gene had 67 % homology with *X. laevis* integrin alpha 2b (accession no. NM_001094754.1). Future work to clone the entire *cd41* gene of FUHEN cells is necessary to determine the function of CD41 in wild tree frog thrombocytes. Additionally, the expression of *cd41* in FUHEN cells definitively concluded that the FUHEN cell line was the first established amphibian thrombocytic cell line.

Some megakaryocytic cell lines have been established in mammals (Tetteroo et al. 1984; Ikebuchi et al. 1998; Takeuchi et al. 1998). More recently, the human iPS-derived megakaryocyte cell line was established. The fact that imMKCLs produce CD42b-positive platelets that can aggregate upon thrombin stimulation suggests that imMKCLs are a promising source of platelets (Nakamura et al. 2014).

### Characteristics of FUHEN cells

The FUHEN cell line has several unique properties. First, FUHEN cells proliferated in suspension culture without adherence to the culture flask or to the stromal cells, which are the main producers of growth factors for hematopoietic cells. Some hematopoietic factors and their receptors were elucidated in *X. laevis*, such as colony stimulating factor-1 and its receptor (Grayfer and Robert 2013), and erythropoietin (Nogawa-Kosaka et al. 2010) and its receptor (Aizawa et al. 2005). Because the FUHEN cells proliferated and grew largely independently of stromal cells, some signaling pathways relevant to proliferation, such as c-MPL signaling, might have been mutated in FUHEN cells.

Interestingly, the FUHEN cells adhered quickly to the petri dish upon the removal of divalent ions (Table 1). Under normal culture conditions, the FUHEN cells did not adhere to the bottom of the flask; however, the cells attached to each other when they proliferated. In amphibians, fishes, reptiles and birds, thrombocytes are directly involved in clotting (Michelson 2013); therefore, the sticky cell characteristics of thrombocytes and the regulation of that stickiness are important for thrombus formation. The cell adhesion molecule integrin usually requires Ca^2+^ for functional activation. Thus, integrin has a Ca^2+^-binding domain, and in zebrafish, for example, CD41 has four Ca^2+^-binding domains (Lin et al. 2005). However, in FUHEN cells, the deprivation of divalent ions induced the rapid adhesion of the cells to the petri dish, while the addition of divalent ions prevented this adhesion (Table 1). In other words, although divalent ions were relevant to this adherence mechanism, the role of Ca^2+^ in switching the adherence mechanism was opposite in tree frogs and mammals. Briefly, the lack of divalent ions quickly activated the adherence mechanism of thrombocytes. This adherence mechanism in particular might enable rapid clotting and the prevention of blood loss in water because the concentration of the divalent ions is lower in fresh water than in serum. Collecting high numbers of circulating thrombocytes from tree frogs is difficult; however, it would be necessary to analyze the effects of divalent ions on the adhesion of these cells in the future. Furthermore, this adhesion mechanism is most likely strictly regulated; otherwise, the thrombocytes would form thrombi in the bloodstream where many thrombocytes are circulating (Hadji-Azimi et al. 1987). The sequestration of divalent ions by EDTA caused the rapid adhesion of the cells to petri dishes, and these adherent cells protruded from the adhesion apparatus (Fig. 5a–d), which must play an important role in rapid adhesion. Meanwhile, with the aggregation of mammalian platelets, shear stress induces tethering and mediates the firm adherence of platelets to vWF, resulting in thrombus formation (Dopheide et al. 2002; Reininger et al. 2006; Maxwell et al. 2007). The protruding adhesion apparatus of the FUHEN cells was not as long as the tether observed in platelets. Phalloidin staining demonstrated that the apparatus was rich in F-actin (Fig. 5e–h). Thus, these results suggest that the apparatus that protruded from adherent FUHEN cells were filopodia.

The FUHEN cells changed their shapes and sizes drastically with growth. The original cell shape was spherical or a small spindle, and the diameter was approximately 15 µm, with a simple ring-like nuclear structure. In contrast to this observation, the later-stage cells were large and oval-shaped, and the long diameter reached more than 40 µm. These cells were complicated, very large and had multi-lobed nuclei (Fig. 4a–c). Furthermore, nuclear phase analysis revealed the existence of cells with more than 8 N ploidy (Fig. 4d). These cells were larger in size than other frog hematopoietic cells. Taken together, these data also indicated that the FUHEN cell line was a thrombocytic cell line. The FUHEN cell line was continuously maintained in suspension culture for more than 8 years. The culture usually contained both normal- and large-size cells, and the large cells were likely mature cells because they had complicated and multi-lobed nuclei. Furthermore, the cloned single, normal-sized cells proliferated, resulting in the production of many large cells. Thus, the culture always contained both immature and mature cells, and the small FUHEN cells were the progenitors of large cells. If the small cells were only progenitor cells, all the cells would have disappeared due to differentiation into large cells; however, the progenitor cells were always present in the culture. This suggested that the progenitor cells have the ability to self-renew. The small FUHEN cells could differentiate into the mature (large) cells and could also produce more of themselves. The identification of the factor that determines the fate of FUHEN cells, whether self-renewal or differentiation, represents future work.

FUHEN cells demonstrated the property of resistance to low temperature. Tree frogs hibernate in winter: the average temperature during hibernation is usually lower than 10 °C, and the 16 °C temperature for culturing was very similar to the average temperature just before the start (November) and end of hibernation (late April). When the FUHEN cells were maintained at 28 °C, they proliferated. No proliferation was observed at 16 °C, and therefore proliferation of the FUHEN may halt at 10 °C. However, although no proliferation was observed at 16 °C, some FUHEN cells could survive at 16 °C for 1 month and reinstated proliferation activity at 28 °C (Fig. 3f). This suggested that the FUHEN cells were briefly sustained at low temperatures by stopping proliferation; therefore, it is possible that naïve thrombocytes in wild frogs also possess this property. Although the precise mechanism was not elucidated in this experiment, it is possible that O-GlcNAcylation was responsible for halting proliferation (Lewis 2013). These findings suggest the possibility that amphibian hematopoietic cells can arrest their cell cycle depending upon the environmental temperature. This property makes it possible to stop the hematopoietic system during hibernation and contributes to the prevention of energy loss, a characteristic that is important for hibernators.

## Conclusions

A new amphibian thrombocytic cell line that resembles the ancestors of mammalian megakaryocytes was established from the tree frog and designated FUHEN. This new cell line was sticky, large, oval-shaped and had stem cell-like qualities. This thrombocyte cell line could provide useful material for studying the function of thrombocytes and the hemostasis mechanisms of amphibians.

## Methods

### Histological analysis and establishment of the cell line

Japanese tree frogs (*Hyla japonica*) were captured in the city of Gosen. The femur was fixed with 10 % formaldehyde solution. Thin frozen sections were prepared and stained with Oil Red O and hematoxylin. An LTBMC was started with minor modification to the murine method (Itoh et al. 1989). Briefly, αMEM and PBS were diluted two-thirds with distilled water to adjust the osmolality. This medium and PBS were used throughout this experiment. The bone marrow cells were flushed into the culture flask (Corning #430372) with αMEM supplemented with 10 % horse serum (HS), 50 ng/mL of testosterone (Wako Pure Chemicals Co. Ltd., Tokyo, Japan) and antibiotics. The cells were incubated in a 5 % CO_2_ low-temperature incubator at 28 °C (model-9100, Wakenyaku, Tokyo, Japan), and half of the medium was changed to fresh medium every 2 weeks. Three months later, the cells were exposed to X-rays (15 Gy), proliferating non-adherent cells were then separated from the stromal cells, and the non-adherent cells were cloned with a limiting dilution method. The non-adherent cell line, designated the FUHEN cell line, was continuously maintained in suspension culture for more than 8 years.

### Effects of temperature on cell growth

The FUHEN cells were seeded in 10 % HS-αMEM in culture flasks at a density of 1 × 10^4^ or 1 × 10^5^ cells/mL and were incubated at 16, 28 or 37 °C. These temperatures were chosen because 16 °C is the temperature just before the start (November) and after the end of hibernation (late April). Due to machine limitations, it was hard to adjust the CO_2_ concentration to 5 % at 10 °C. Therefore, 16 °C was chosen. The number of living cells was counted every week for 1 month. In some experiments, the cells were shifted to 28 °C from 16 or 37 °C after several weeks of culture to check the reproductive capacity of the cells.

### Characteristics of the cell line

The cells were fixed with 10 % formaldehyde in PBS for 30 min. Then, the cells were permeabilized with 0.1 % Triton X-100 in PBS and stained with DAPI and were then observed with a confocal microscope (Carl Zeiss Microscopy, LSM780) or a fluorescence microscope (Olympus, IX71). In another experiment, the proliferating cells were fixed with 70 % EtOH for 24 h. Then, the cells were washed with PBS and treated with RNase (20 µg/mL) for 4 h at 4 °C. The cells were then stained with propidium iodide (PI) just before analysis using a FACSAria II (BD, Tokyo Japan).

### Adhesion test

The FUHEN cells were washed two times with 1 % HS-αMEM and were suspended with 10 %HS-αMEM, 0.02 % EDTA/PBS, PBS or 0.1 mg/mL MgCl_2_·6H_2_O and 0.1 mg/mL CaCl_2_·2H_2_O/PBS, 10 % HS-PBS or 10 % HS-EDTA/PBS. Then, the cells were seeded in four petri dishes. After 5 min of incubation at room temperature, the dishes were washed two times with PBS and fixed. Then, the cells were stained with hematoxylin or May-Grünwald Giemsa, and photographs were taken. The number of attached cells per area was counted using ImageJ software. In another experiment, the cells were fixed with 10 % formaldehyde in PBS for 12 h at 4 °C. Then, the cells were treated with 0.1 % Triton X-100 for 5 min at room temperature. The cells were washed with PBS and stained with the actin stain 555 Fluorescent Phalloidin (Cytoskeleton, Inc., Denver, USA) and DAPI, according to the manufacturer’s instructions.

### Partial cloning of *cd41*

Degenerate oligo DNA primers were designed (Lin et al. 2005). The sequence for the forward primer was 5′-GGMCCYCCKGGHAGCYACTTTGGNTT, and the reverse primer was 5′-TTCCANTGMTGHAAKGGBGCACA. Briefly, cDNA was transcribed from total RNA of the FUHEN cells using the ReverTra Ace qPCR RT Master Mix with gDNA Remover (TOYOBO Co. Ltd, Tokyo, Japan). PCR was performed using *Ex Taq* DNA polymerase as described previously (Sugimoto and Jiang 2008), and the amplified PCR product was sequenced.

## Additional files

Additional file 1:
**Figure S1.** Surviving FUHEN cells without a medium change. FUHEN cells were cultured at 28°C without a medium change. Some FUHEN cells survived (black arrow). These cells started to proliferate when the cells were suspended in fresh medium (data not shown). Additional file 2:
**Movie S1** and **Movie S2.** Fine structure of FUHEN cells. FUHEN cells were stained with DAPI and were observed with a confocal microscope. A multi-lobed, very large nucleus was observed in a cell. A ring-like nuclear structure was also observed. Movie S1a, S1b and Movie S2a, S2b also demonstrate the fine structure of other FUHEN cells.
