# Supplementary figures and images for: Establishment of a sticky, large, oval-shaped thrombocyte cell line from tree frog as an ancestor of mammalian megakaryocytes
Source: Springerplus. 2015 Aug 25;4:447. doi: 10.1186/s40064-015-1237-7 (PMC4547970; doi:10.1186/s40064-015-1237-7)

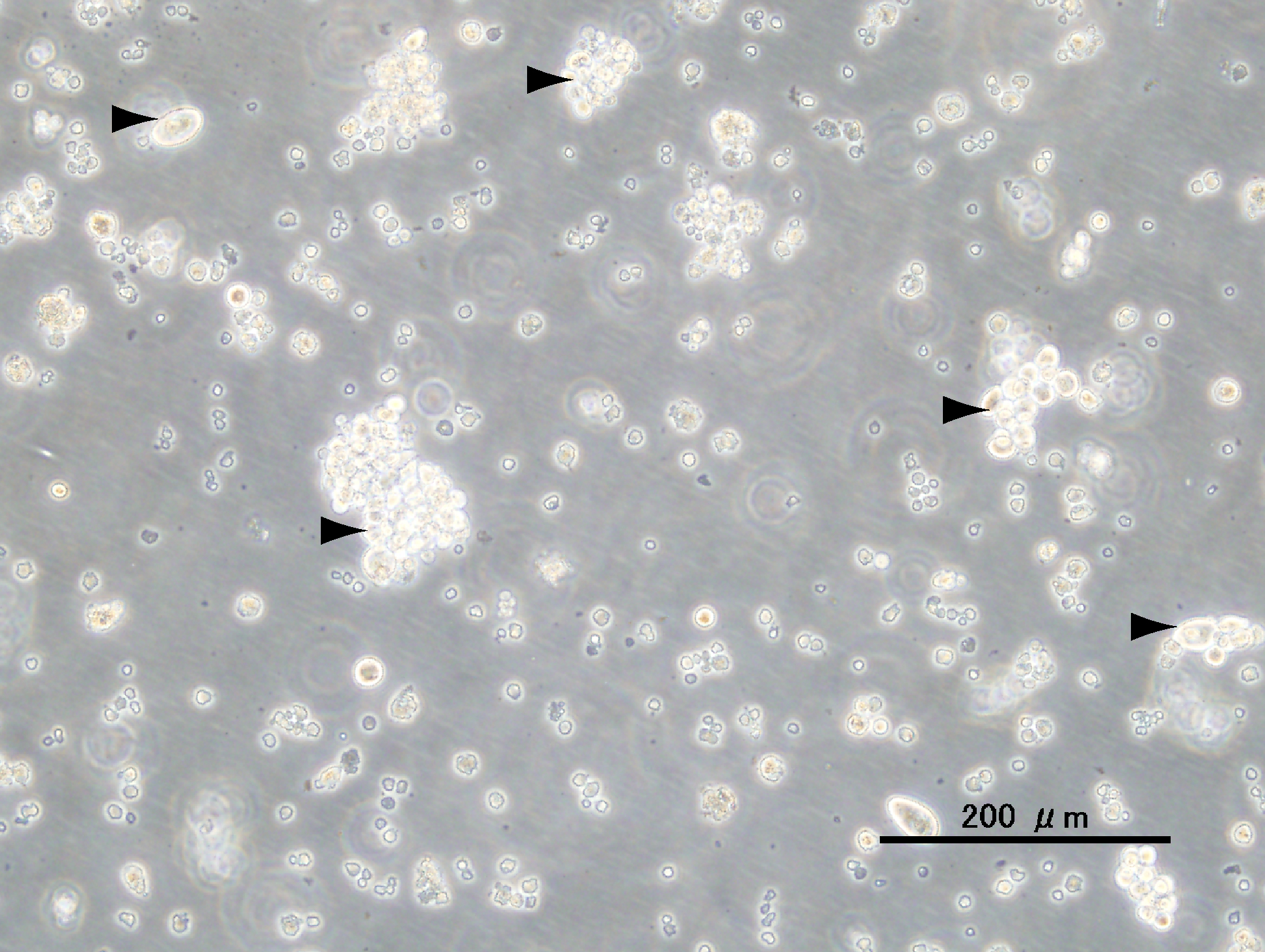

Supplement: Additional file 1: — Figure S1. Surviving FUHEN cells without a medium change. FUHEN cells were cultured at 28°C without a medium change. Some FUHEN cells survived (black arrow). These cells started to proliferate when the cells were suspended in fresh medium (data not shown). [file 40064_2015_1237_MOESM1_ESM.jpg]
